# Perceptions about the Self-Learning Methodology in Simulated Environments in Nursing Students: A Mixed Study

**DOI:** 10.3390/ijerph16234646

**Published:** 2019-11-22

**Authors:** José Luis Díaz Agea, Antonio Jesús Ramos-Morcillo, Francisco José Amo Setien, María Ruzafa-Martínez, César Hueso-Montoro, César Leal-Costa

**Affiliations:** 1 Faculty of Nursing, Catholic University of Murcia, 30107 Guadalupe, Spain; jluis@ucam.edu; 2 Faculty of Nursing, University of Murcia, 30100 Espinardo, Spain; cleal@um.es; 3 Faculty of Nursing, Cantabria University, 39005 Santander, Spain; franciscojose.amo@unican.es; 4 Faculty of Health Sciences, University of Granada, 18016 Granada, Spain; cesarhueso@ugr.es

**Keywords:** self-directed learning, peer learning, clinical simulation, nursing students

## Abstract

The self-learning methodology in simulated environments (MAES©) is an active method of education. The aim of this study was to analyze the perceptions and opinions of undergraduate and graduate nursing students about the self-learning methodology in simulated environments. A mixed, cross-sectional, descriptive study based on a survey tool made ad hoc (quantitative approach) and an open questionnaire (qualitative approach) was carried out. A sample of 149 undergraduate and 25 postgraduate nursing students were tested. The score was high for all the variables of the questionnaire analyzed: for perception of simulation performance, M = 73.5 (SD = 14.5), for motivation, M = 23.9 (SD = 5.9), for the opinion about facilitators, M = 25.9 (SD = 4.5), and for the promotion of team work, M = 16.9 (SD = 3.4). Five dimensions were identified and evaluated in the qualitative research. The students were pleased with MAES© and had a positive perception, since they considered that MAES© increased their learning.

## 1. Introduction

There is currently a wide range of possibilities in learning methods for health professionals [1,2,3,4]. With the traditional teaching methodologies, the students had a passive role [5]. In addition, the old teaching methods failed to take sufficient account of patient safety, and health professionals carried out their supervised practices after a theoretical training or, in any case, with low-fidelity simulations. Subsequently, to improve patient safety and allow students to take an active role in learning, new methodologies based on clinical scenario simulations emerged [6].

In theory, passive learning is gradually being transformed into active learning for students in Europe (following the guidelines of the European Higher Education Area) [7]. However, in the reality of nursing learning, master classes and lessons explained by an expert (the lecturer) still persist in our context as the main way of acquiring knowledge. Theoretical contents are explained in class and, on the other hand, practical skills are acquired during skill-based seminars, performing simulations, or real clinical practices.

At the Catholic University of Murcia, Spain, (UCAM), nursing students learn with different simulation styles: simulation-based learning (SBL) in which facilitators design the scenarios and program the competencies to be acquired, and self-learning methodology in simulated environments. MAES is the Spanish acronym for self-learning methodology in simulated environments (Metodología de Autoaprendizaje en Entornos Simulados) [8,9] that was implemented in the academic year 2013–2014. Currently, MAES© is used to teach nursing students at some universities in Spain and it is being considered for teaching in other countries (like Brazil, Portugal and Norway).

MAES© is structured through the synergistic union of other active learning methodologies such as problem-based learning (PBL), SBL, peer education, and self-directed learning. Through the self-directed learning that MAES© promotes [10], students develop and acquire competences that they voluntarily choose to learn and which are difficult to include in traditional curricula, such as communication skills, reasoning skills, advanced technical skills, or empathy. This is possible when students are driven by an intrinsic motivation and greater autonomy in their skill acquisition process. MAES© was designed for students to develop analytical skills, fostering thinking and teamwork skills [11]. With this methodology, students should be the protagonists of the whole learning process, which is ultimately a creative process, from the choice of the case they want to study and design to the end of the simulated experience and debriefing, establishing the conditions for a greater experience of reflection.

MAES© consists of six elements [9] and is structured in a minimum of two sessions. In the first session, a suitable and psychologically safe working environment is created, and then operational work teams are established. The teams are autonomous and have a defined group identity (Element 1). Each team will freely choose a topic that will serve as inspiration for the design of a simulation scenario (Element 2). The topics are based on a problem that is related to the nursing profession or the subject of the course, which is presented in the form of news or videos drawn from reality or fiction and which the facilitator uses as a "bait" or "hook". After the case is chosen, a joint brainstorming session is held in which the students, by teams, decide what they know about the subject and then what they want to learn from it, as well as the skills (clinical and non-technical) they would like to acquire with the case they have chosen (Element 3). 

Once the session is over, each team commits to designing a scenario, which another team will experience in the simulation room within a week. In addition, they are also committed to seeking information about what "we want to know" and skills (Element 4).

In a second session, another team (different from the one that has designed the case) will enter the high-fidelity simulation room and will have a simulation experience with a simulated patient, a mannequin or a standardized patient (Element 5). The simulation will be followed by a structured debriefing (Element 6) [12]. Debriefing is considered as a key component of the MAES© methodology [13]. In addition to analyzing the performance of the participants in the scenario, the students explain what they learned during the design of the case and look for evidence of the learning objectives, which would be the points they agreed upon in the first session [9]. Therefore, debriefing in the MAES© methodology includes an expository phase in which the team that designed the scenario would teach other students the skills that the whole group chose to learn from the case. The students commit themselves to support the debriefing expository phase with reliable and quality scientific evidence.

Each session deals with three scenarios and involves six work teams (three teams design and present and three teams experiment and reflect together). An average of 12–16 students participate in the MAES simulation sessions (distributed in six work teams). In each session, three scenarios are worked on with an average duration of four hours (including breaks).

Leaving the responsibility of learning to the students themselves is a challenge. In order to do this, the educator needs to stand back, abandon the role of transmitter of knowledge, and become a facilitator of learning. The controversy surrounding self-learning and peer-to-peer learning (based on the transmission of knowledge among equals) has been a subject of debate [14]. Despite being an important complement to teaching time, some believe that self-learning by itself cannot replace guided learning [15]. MAES© was designed with the aim that students learn in a collaborative way, which should have a subjective impact on communication skills and possibly increase teamwork skills [11]. When compared to SBL, MAES© grants students a better degree of performance in learning with simulation [16].

In this work, we wanted to know the opinions and perceptions of the students, both undergraduate and graduate in nursing, in relation to the use of MAES© as a learning strategy. Likewise, we intended to provide the perspective of students who have been trained with MAES©. It should be noted that the students themselves are responsible for their learning and for transferring it to their peers, so studying their point of view about MAES© is important, to know, in a deeper way, its characteristics and the pedagogical impact it has on students.

Above all, we were interested in asking about the students’ perception of simulation performance. We also wanted to know the level of satisfaction of the participants in MAES© sessions. Another objective was to determine the motivation of the students and the promotion of teamwork, as well as the students’ opinions about the facilitators.

## 2. Materials and Methods 

### 2.1. Study Design

The present study was based on a mixed, cross-sectional, and descriptive method and examined the subjective pedagogical impact (students´ perception and satisfaction in different areas) of the MAES© methodology in undergraduate and postgraduate students who learnt using it at the Catholic University of Murcia, Spain.

### 2.2. Participants and Research Context

The study was conducted with a sample of fourth-year students of the nursing degree (undergraduate) and of students of the emergency nursing master’s degree (postgraduate) at UCAM from 2015 to 2018 (*N* = 330). Finally, a sample of 174 students was obtained. Non-probabilistic convenience sampling was used. An on-line questionnaire was offered to all enrolled students in the course (4th grade and master´s degree), and the students could respond voluntarily once the course was completed.

### 2.3. Data Collection and Analysis

The data collection was carried out through an on-line survey designed ad hoc with 21 items, whose responses, measured with the Likert scale, expressed the students’ level of agreement with statements (quantitative method) and through an open question where each student felt free to write his or her personal opinion about MAES© (qualitative method). 

The dimensions and items of the questionnaire were first agreed upon by the research team and then validated (content validity) by an expert committee following the recommendations of the literature. Polit and Beck [17] defined content validity as "the degree to which a sample of items, taken together, constitute an adequate operational definition of a construct".

The expert panel consisted of five MAES© simulation facilitators with clinical experience and doctoral degrees in different fields (1 PhD in nursing, 2 PhD in psychology, 1 PhD in anthropology, and 1 PhD in medicine). The design phase of the questionnaire was carried out during July 2014, and an adequate content validity index (CVI = 0.9) was calculated before proceeding to use the survey. 

Finally, a questionnaire with 5 dimensions was designed. Each dimension was explored with items, each of which would be assessed with a Likert numerical scale from 1 to 10 (measuring the degree of agreement, with 1 being total disagreement, and 10 absolute agreement). The dimensions were the following:

1. Perception of simulation performance. This dimension refers to: how MAES© affects the learning capacity of the students; how MAES© influences the experience of scenario simulation; the deepening of the knowledge of the clinical situation when the students design the case; whether the debriefing is enriching. It contains 9 items (maximum score 90, minimum score 9).
2. Motivation. This dimension measures the effects that both the group identity (generated in the group dynamics of the first session) and the voluntary choice of a scenario have on the motivation of the students. It consists of 3 items (maximum score 30, minimum score 3).
3. Satisfaction. This dimension evaluates the satisfaction of the students in using the MAES© methodology. It consists of 4 items (maximum score 40, minimum score 4).
4. Opinion about the MAES© facilitators. This dimension determines the student’s perception of the educators’ preparation in this methodology, as well as the role they play. It contains 3 items (maximum score 30, minimum score 3).
5. Promotion of group work. This dimension measures the impact of collective work on the perception of the students. It includes 2 items (maximum score 20, minimum score 2).

The reliability of the scale was analyzed according to the internal consistency of Cronbach’s alpha [18]. SPSS v23 (statistical package for social sciences, published in August 2014, IBM SPSS Statistics for Windows version 23.0, IBM Corp., Armonk, NY, USA) was used to analyze the data.

With respect to the open-ended question, the following statement was proposed and answered in writing at the end of the questionnaire: "Write in a few lines your sincere opinion about the simulation with MAES©, positive issues or improvements that you think are convenient if there were any." In order to classify and present the results on this question, a conventional content analysis was carried out. This analysis consisted of reading the data obtained, word by word, highlighting the exact words in the text that captured ideas or key thoughts, in order to translate them into codes, that is, to join several of these ideas or thoughts in only one. These codes, according to the relationship they had with each other, were classified by categories, and these, in turn, were organized into larger dimensions [19].

### 2.4. Ethical Considerations

For this research, ethical principles were followed, and people’s rights were not violated. The students were surveyed voluntarily, obtaining their consent in advance. Through announcements on the virtual campus, they were informed about the confidentiality of the answers to the test and their anonymity. For the literal transcription of the qualitative results, an alphanumeric coding/encryption system was followed (S1D = Student 1 undergraduate, S3M = Student 3 of the master’s degree). The research was approved by the Ethics Committee of the Catholic University of Murcia and evaluated with a favorable opinion (reference number: 5939 02/02/2016).

## 3. Results

The sample finally consisted of 149 fourth grade nursing students (85.6%) and 25 students of the emergency nursing master’s degree (14.4%). Most of them were female (71.3%), and the mean age of the participants was 26.7 (SD = 6.7) years.

### 3.1. Questionnaire Results

The students (*n* = 174) scored on the dimensions as follows: 1. Perception of simulation performance, M = 73.52 (SD = 14.56); Motivation, M = 23.97 (SD = 5.97); Satisfaction, M = 30.98 (SD = 6.48); Opinion about the MAES© facilitators, M = 25.98 (SD = 4.55); Promotion of teamwork, M = 16.91 (SD = 3.49). Adequate internal consistency of all dimensions was demonstrated by analyzing the Cronbach alpha between the items of each dimension (Table 1).

### 3.2. Qualitative Results

After performing a content analysis of the students’ free response to the open-ended survey question, the following dimensions appeared:

1. Operational aspects: Our students extend the positive characteristics of the MAES© methodology, adding that it is demanding “… it is a demanding methodology for the student, which I find very positive…” (S1G). They agree that it increases the safety and confidence of students, so one can work in a safe learning environment. They also think that MAES© is an incentive to practice teamwork. “I think it’s a dynamic way to learn and work as a team” (S18G). Likewise, the choice of cases by the students themselves is very enriching, “… to work from scratch with a case that we have previously chosen.” (S13G). In addition, the students feel that MAES© is a methodology that increases learning on a personal level, “improves self-learning promotion” (S49G).

2. Emotional aspects: In this regard, the participants agreed that it is a way of working that decreases the anxiety that is inherent in the simulation, which helps to work more relaxed, and that it is active, dynamic, or enjoyable. "From the point of view of the nerves, with this type of simulation, we are more relaxed…" (S13G), “… we are not so nervous…” (S4G), “The MAES© methodology is very creative and participatory” (S63G).

3. Facilitation: On the other hand, students refer to the importance of the facilitator’s role as a motivating and stimulating element in the simulation sessions: “I think the instructor plays a fundamental role…” (S3G), “… with the professor’s support…” (S13G), “… contributing their knowledge and guidance… (S71G) ”, “Facilitators’ contributions are fundamental” (S155G).

4. Debriefing development: Students considered that debriefings were more enjoyable and participative: "we discuss the cases in a fun way" (S4G). There is a greater involvement by respondents “… we are more… involved” (S137G), “… you get involved…” (S138G), “… learning based on scientific evidence…” (S190M).

5. Areas of improvement: Some students expressed the need to combine MAES© with other simulation methodologies more restrictive or based on low fidelity (learning technical skills). Others added that high-fidelity simulation was slightly uncomfortable for them: “Working under pressure and being observed limits a lot the performance and does not accurately reflect what you are capable of doing.” (S80G). Other respondents expressed as an inconvenience the limitations inherent in teamwork, i.e., disagreements between the team members or passive colleagues, whose performance is lower but “… will get the same rating as the rest” (S87G).

## 4. Discussion

The results corroborate the good acceptance of active learning methodologies when they focus on students rather than on educators, as some studies in different pedagogical areas have shown [20,21]. Regarding the perception of simulation performance recognized by the students, it is necessary to highlight the high score of the aspects related to the improvement of the learning capacity (M = 8.49) and the stimulation of creativity (M = 8.41). This may be because MAES© is a pedagogical model based on problem-solving, which would pose a challenge for the students. The qualitative results indicated that the student’s confidence was greater than with traditional learning, which would contribute to stimulate their self-esteem as active participants in the teaching/learning process.

As for the motivational aspects, these go hand in hand with an intrinsic appreciation of them, so that the most valued item was the autonomy or freedom of choice of the skills to acquire (M = 8.33), being the main stimulus for learning, instead of qualifications (extrinsic motivation).

The "satisfaction" dimension was well valued, especially when comparing the degree of satisfaction with the MAES© methodology (M = 8.57) with satisfaction with other simulation methodologies (SBL) for which the mean satisfaction score was lower (M = 7.18). Another study [22] revealed that a high level of student satisfaction contributes to a better use of learning. This study found three factors that influenced student satisfaction. The first concerns the instructor’s performance, the second is the student’s commitment to learning, and the third is the course’s policies. In this sense, the results coincide with this work on MAES©, since the students who responded expressed high levels of satisfaction with a learning environment that requires the instructor and the students to take responsibility for learning together and in a bidirectional way (two-way learning). The results also support that teaching performance contributed crucially to student’s subjective perceptions of learning, especially with regard to the qualification of the facilitators (M = 9.02), which was the best-rated item in the survey, as well as to the role of the instructor in motivating students (M = 8.66). The qualitative results explain that the role of the facilitator was crucial, in spite of the fact that the facilitator was not the main source of transmission of knowledge but was only required to facilitate its acquisition.

The promotion of teamwork and a reinforced group identity are key characteristics in the pedagogical context [23] and are the main elements of MAES© that promoted a good evaluation of this dimension.

Regarding the dimension of emotional and behavioral aspects, the students considered that with MAES© they learned in a more relaxed way and with less anxiety. It appears that choosing the topics to be addressed, as well as the aspects to learn can help to reduce students’ uncertainty and nervousness. However, the aspect of MAES© that should be improved according to the students was "feeling observed", which they considered as a handicap, preventing them from performing the simulation in a natural way. We understand that this is a cross-cutting aspect of clinical simulation and not a particular feature of MAES© [24]. Another interesting aspect to emphasize is that, when working in a team, students should have certain interpersonal skills that facilitate learning with MAES. However, if any member of the team is not up to the task, it may lead to the failure of the rest of the team. We believe that the fear of obtaining a low grade because of a student with a poor simulation performance is one of the reasons why students identified this weak point in the MAES© methodology in our study.

The students, in general, were satisfied with this methodology because it presents some advantages that were very appreciated by them and that have also been shown in other studies [10,16,25,26]. The freedom of choice in learning allows students to practice skills that they have not been able to perform during clinical practice, or at least not often enough, and that, in this case, they can practice in a safe environment. In relation to debriefing with MAES©, the respondents considered that it was more fun and participatory than debriefing with other simulation methods. Working with MAES© enabled student empowerment and self-management of the reflective process that influenced the way debriefing developed. However, the students agreed that debriefing is a process that must always be guided by a facilitator.

### Limitations

In the present work, no assessment of test–retest or temporal stability was conducted, so it was not possible to verify this aspect of reliability. Another limitation could be the voluntary nature of the survey response, which could lead to a non-response bias or voluntary effect, which, in turn, may lead to the lack of knowledge of many students’ opinions, including disagreements with the MAES© method.

## 5. Conclusions

The surveyed students were satisfied with the MAES© methodology. The subjective impact of MAES© on the students was clearly positive. The students recognized numerous advantages in this methodology, such as the reduction of the anxiety related to the simulation and a more dynamic and active way of learning. The participants considered that this methodology increased motivation and promoted teamwork. They also perceived that they learned much more and better by working in teams and with a defined group identity. The students considered the facilitators as essential elements in the process of acquiring competences with MAES©. Some minority opinions were obtained on aspects to be improved in learning with MAES© and should be taken into account. These aspects are related to the stress of simulation as a learning method and to potential problems derived from teamwork (when a team member is a student with a poor simulation performance, and this influences the overall performance of the team).

## Figures and Tables

**Table 1:** Statistical description of the dimensions and items of the questionnaire, as well as Cronbach alpha of the dimensions. Self-Learning Methodology in Simulated Environments (MAES©)

Dimensions and items *(n = 174)*	*M* *ean*	*Standard Deviation*	*Cronbach Alpha*
*Dimension: 1 Advantages of MAES©*	73.52	14.56	*0.93* *9*
*The MAES methodology improves my ability to learn*	8.49	1.63	
*I feel more confident with MAES than with other methodologies when I practice with cases in the simulation room.*	7.78	2.198	
*I think I acquire more knowledge when I learn with MAES than with other high-fidelity simulation methodologies*	8.07	2.002	
*The debriefing is richer, and I prepare it better when working with the MAES method*	7.92	2.176	
*Although it takes longer to prepare the case, I go deeper into the knowledge of the clinical situation*	8.20	2.07	
*Learning with MAES stimulates my creativity and my ability to investigate and solve problems*	8.41	1.859	
*I believe that MAES makes me feel more prepared to face a situation in reality, although I have previously worked in simulation*	8.31	1.964	
*My degree of responsibility in my own learning is high, so I learn more*	8.14	1.832	
*I think that my learning is more efficient with MAES than with other simulation methodologies*	8.26	1.952	
*Dimension: 2 Motivation*	23.97	5.974	*0.838*
*The freedom in the choice of cases stimulates my learning, and my role is more active*	8.33	1.872	
*I feel more motivated with MAES than with other clinical simulation methodologies*	8.11	2.053	
*Promoting group identity in MAES sessions (forming teams with a nickname and a group spirit, for example) increases the motivation of the students*	7.53	2.66	
*Dimension: 3 Satisfaction*	30.98	6.480	*0.740*
*The effort that the MAES methodology requires from the students is worth it*	8.56	1.867	
*I would prefer to learn only with the MAES simulation methodology*	6.67	2.862	
*Rate your level of satisfaction with the MAES methodology*	8.57	1.735	
*Rate your degree of satisfaction with other simulation learning methodologies that you have experienced in your training (excluding MAES)*	7.18	2.0	
*Dimension: 4 Opinion about the MAES facilitators*	25.98	4.552	*0.780*
*I consider that the facilitators are sufficiently prepared to work with MAES learning groups*	9.02	1.528	
*The responsibility for learning lies with the students, and the facilitator plays a very important but secondary role*	8.29	2.057	
*I consider that the role of the teacher is crucial in the initial motivation of the simulation groups that work with MAES, rather than in the transmission of knowledge*	8.66	1.839	
*Dimension: 5 Promotion of teamwork*	16.91	3.489	*0. 826*
*The process of selection, design, and preparation of the case encourages teamwork*	8.53	1.880	
*I believe that, thanks to the group work promoted by MAES, the group acquires more knowledge than with other simulation methods*		*1.900*	
